# Advances in Alzheimer’s Disease-Associated Aβ Therapy Based on Peptide

**DOI:** 10.3390/ijms241713110

**Published:** 2023-08-23

**Authors:** Cunli Wang, Shuai Shao, Na Li, Zhengyao Zhang, Hangyu Zhang, Bo Liu

**Affiliations:** 1 School of Biomedical Engineering, Faculty of Medicine, Dalian University of Technology, Lingshui Road, Dalian 116024, China; wangcunli@dicp.ac.cn (C.W.); shaos@dlut.edu.cn (S.S.); lina316@dlut.edu.cn (N.L.); zhengyaozhang@dlut.edu.cn (Z.Z.); hangyuz@dlut.edu.cn (H.Z.); 2 Liaoning Key Lab of Integrated Circuit and Biomedical Electronic System, Dalian University of Technology, Dalian 116024, China; 3 School of Life and Pharmaceutical Sciences, Dalian University of Technology, Panjin 124221, China

**Keywords:** Alzheimer’s disease, peptide-based therapy

## Abstract

Alzheimer’s disease (AD) urgently needs innovative treatments due to the increasing aging population and lack of effective drugs and therapies. The amyloid fibrosis of AD-associated β-amyloid (Aβ) that could induce a series of cascades, such as oxidative stress and inflammation, is a critical factor in the progression of AD. Recently, peptide-based therapies for AD are expected to be great potential strategies for the high specificity to the targets, low toxicity, fast blood clearance, rapid cell and tissue permeability, and superior biochemical characteristics. Specifically, various chiral amino acids or peptide-modified interfaces draw much attention as effective manners to inhibit Aβ fibrillation. On the other hand, peptide-based inhibitors could be obtained through affinity screening such as phage display or by rational design based on the core sequence of Aβ fibrosis or by computer aided drug design based on the structure of Aβ. These peptide-based therapies can inhibit Aβ fibrillation and reduce cytotoxicity induced by Aβ aggregation and some have been shown to relieve cognition in AD model mice and reduce Aβ plaques in mice brains. This review summarizes the design method and characteristics of peptide inhibitors and their effect on the amyloid fibrosis of Aβ. We further describe some analysis methods for evaluating the inhibitory effect and point out the challenges in these areas, and possible directions for the design of AD drugs based on peptides, which lay the foundation for the development of new effective drugs in the future.

## 1. Introduction

Alzheimer’s disease (AD), the most common dementia, is expected to affect about 150 million individuals worldwide by 2050, resulting in considerable personal and public health burdens [1,2]. The clinical features of AD are mainly characterized by short-term memory impairment, aphasia, apraxia, agnosia, and executive dysfunction [3,4,5]. The main biological feature of AD is the presence of β-amyloid (Aβ)-containing plaques in the hippocampus and cortex of the brain and tau-containing neurofibrillary tangles in nerve cells [6,7]. Both preclinical and clinical studies regard the misfolding of Aβ (mainly containing Aβ40 and Aβ42) and Aβ aggregation-induced cascades, including oxidative stress and inflammation, as the critical factors in the progression of AD [8,9,10,11]. A recent study indicates that the clinical failure rate of drugs intervening AD has been as high as 99.6% in more than 100 years since AD was discovered [12]. The failure of drugs to improve cognitive impairment in patients is mainly attributed to an inappropriately chosen target, drug-associated side effects, and neuroinflammatory responses [11,13]. Aducanumab, which has obtained accelerated approval from the FDA for AD treatment, is used to eliminate aggregated soluble and insoluble forms of Aβ. However, it is still controversial and needs further patient assessment [14]. These failures remind us that breaking the dilemma of AD treatment needs effective new drugs and therapies that have a strong inhibitory effect on the target, low immunogenicity, and excellent biocompatibility.

Over the recent decade, peptide-based therapeutics have been up and coming and risen to prominence. For example, enfuvirtide is a 36 amino acid biomimetic peptide used in the treatment of HIV-1 [15]. Ziconotide is used to manage severe chronic pain [16,17]. All these peptide drugs have already been used in a wide range of therapeutic areas, such as urology, respiratory, pain, oncology, metabolic, cardiovascular, and antimicrobial applications [18,19,20,21,22]. To date, there are more than 60 FDA-approved peptide drugs for treating diseases, and more than 400 therapeutic peptides are currently in the clinical stage [23]. Compared with other small molecules and antibodies, peptides could be easily modified and infiltrated into tissues and cells. Furthermore, peptides have excellent biocompatibility with low immunogenicity in vivo. The above robust properties enable peptides to be appealing candidates for AD treatment.

Peptides are usually composed of well-ordered amino acid chains with molecular weights of 500–5000 Da [24]. Peptides can be obtained through various methods, and their structure and function are determined by their amino acid sequences. While chirality, an inherent property of peptides, has been recognized as a vital factor in peptide application [25], accordingly, some studies mainly focus on investigating the chirality of amino acids or peptide-based materials on Aβ aggregation since chiral amino acid- or peptide-based materials can recognize biomolecules and perform essential physiological activities [26]. On the other hand, some researches screen specific peptides against target molecules such as Aβ through phase display. Subsequently, the peptides obtained using this technology should be verified for high affinity to Aβ and thus applied to modulate Aβ conformation and aggregation. Moreover, some strategies have been conducted to design functional peptides, which are used to inhibit Aβ aggregation, based on amyloid fibrosis core sequence and the structure of Aβ. This review summarizes the AD-related therapies based on peptides and highlights how to design functional peptides, providing a new perspective on the design of AD drugs based on peptide inhibitors.

## 2. Design of Chiral Amino Acid- or Chiral Peptide-Based Inhibitors

In living systems, chiral biomolecules are endowed with inherent stereoselectivity. For instance, DNA and RNA only contain D-sugars, and most proteins are composed of L-amino acids. Regularly, only one of the enantiomers is pharmaceutically active, while the other is inactive or exerts severe side effects [27,28]. Recent studies have proved that amino acid chirality could determine the folding of the peptide backbone, specific binding with guests through stereoselective hydrogen bonding interactions, and even the bio-function of proteins in vivo [29,30,31]. Thus, investigating the modification of chiral amino acid or peptides and their inhibitory effect on Aβ aggregation has become the mainstream of new drug development in AD treatment.

**Figure 1:**
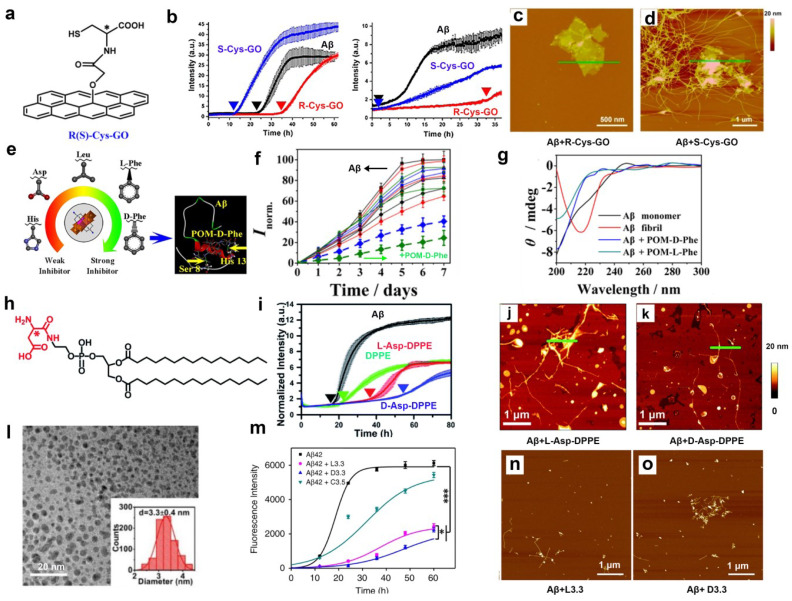
Chiral amino acids or peptides modified interfaces and their inhibitory effect on Aβ aggregation. (**a**) The structure of graphene oxide (GO) modified with R(S)-cysteine (R(S)-Cys-GO). (**b**–**d**) The effect of R(S)-Cys-GO on Aβ aggregation. ThT-monitored kinetic curves (**b**) and AFM images (**c**,**d**) for fiber formation of Aβ or Aβ treated with R(S)-Cys-GO. Different colored arrows represent the initial aggregation time of Aβ. The corresponding sectional profile along the green line is applied to show the lower panel of each image. (**e**) Different chiral amino acid-modified polyoxometalates (POMs). (**f**) Aggregation kinetics of Aβ monitored using ThT fluorescence with the addition of different chiral-modified POMs. (**g**) The inhibitory effect of POM-D-Phe and POM-L-Phe on Aβ aggregation monitored using CD. (**h**) The molecular structure of L(D)-Asp-DPPE. (**i**–**k**) The effect of L(D)-Asp-DPPE on Aβ aggregation through ThT fluorescence (**i**) and AFM images (**j**,**k**). The colored arrows and green line represent the same meaning described above. (**l**) Morphology and size of L- and D-glutathione-modified gold nanoparticles (L3.3 and D3.3). (**m**–**o**), The effect of L3.3 and D3.3 on Aβ aggregation through ThT fluorescence (m) and AFM images (**n**,**o**). * *p* < 0.05, *** *p* < 0.001, indicating a significant difference. (**a**–**d**) Reproduced with permission from [32], Copyright 2014, American Chemical Society. (**e**–**g**) Reproduced with permission from [38], Copyright 2019, American Chemical Society. Images of (**h**–**k**) are reproduced from [41] with permission from Copyright 2020, Royal Society of Chemistry. (**l**–**o**) Reproduced with permission [42]. CC BY (http://creativecommons.org/licenses/by/4.0/), accessed on 22 September 2020.

For instance, Sun et al. used cysteine enantiomer-modified graphene oxide (GO) (Figure 1a) to regulate Aβ40 aggregation in vitro [32]. As shown in Figure 1b, it was found that R-cysteine-modified GO largely inhibited Aβ fibril formation as evidenced by the standard thioflavin-T (ThT) binding assay [33] at the beginning of incubation with Aβ40, but S modification promoted this fibril formation process. Compared with S modification, the application of R-cysteine modification still inhibited Aβ40 aggregation even though it was added after Aβ40 was aggregated for 10 h. Furthermore, atomic force microscopy (AFM) [34] results confirmed previous findings on the effectiveness of R-form of cysteine in GO (Figure 1c,d). Interestingly, the inhibitory effect was highly related to the distance between chiral moieties and GO surface, a short distance contributing to the better inhibitory effect on Aβ40 aggregation. Similar results have been observed on R-cysteine- and S-cysteine-modified silicon oxide interfaces [35]. Polyoxometalates (POMs) have been introduced in numerous fields, such as biochemical catalysts, anti-HIV drugs, and antibiotic agents [36]. Furthermore, POMs can recognize target biomolecules through metal substitution and organic derivatization [37]. Therefore, Qu et al. synthesized a series of D- and L-amino acid-modified POM derivatives, including positive D-His and L-His, negative D-Glu and L-Glu, and hydrophobic D-Leu, L-Leu, D-Phe, and L-Phe, to modulate Aβ40 aggregation (Figure 1e) [38]. ThT results indicated that Phe-modified POMs, particularly D-Phe-modified POMs, showed a stronger inhibitory effect on Aβ40 aggregation than other amino acid-modified POMs (Figure 1f). Circular dichroism (CD) spectra [39] were further applied to detect the conformation change of Aβ40 after being treated with Phe-modified POMs. The result concluded that D-Phe-modified POMs could keep Aβ40 in a monomer state, thus inhibiting Aβ40 aggregation (Figure 1g). They further explained the strong inhibitory effect of D-Phe modified POMs because they have a stronger binding affinity to Aβ40 than other derivatives. Recently, liposomes have been considered promising and versatile drug vesicles [40]. Qing et al. fabricated a pair of chiral liposomes to inhibit Aβ40 aggregation [41]. Specifically, L- and D-aspartic acid-modified 1,2-dipalmitoyl-sn-glycero-3-phosphoethanolamine (L-/D-Asp-DPPE, shown in Figure 1h) was synthesized to construct chiral phospholipid bilayers. D-Asp-DPPE liposomes had a strong inhibitory effect on Aβ40 aggregation compared with L-Asp-DPPE liposomes through ThT and AFM experiments (Figure 1i–k). The affinity tests were also conducted to prove that D-Asp-DPPE liposomes have a higher affinity for Aβ40 than L-Asp-DPPE liposomes, thus having a stronger inhibitory effect on Aβ40 aggregation. Gold nanoparticles provided a platform for modifying peptides and studying peptide–peptide interactions. Tang et al. designed and prepared 3.3 nm L- and D-glutathione-stabilized gold nanoparticles (denoted as L3.3 and D3.3, respectively) shown in Figure 1l [42]. D3.3 possessed a more significant binding affinity to Aβ42, and thus D3.3 gave rise to a stronger inhibitory effect on Aβ42 fibrillation (Figure 1m–o). The author also proved that D3.3 had an excellent therapeutic effect on AD model mice. The above studies demonstrate that chiral amino acid- or peptide-modified interfaces have different effects on Aβ aggregation, and they provide some potential therapeutic approaches for AD treatment. Chiral peptides, which inhibit Aβ fibrillation, can be obtained via a mirror phage display and are reviewed later.

## 3. Design of Peptide through Phage Display

**Figure 2:**
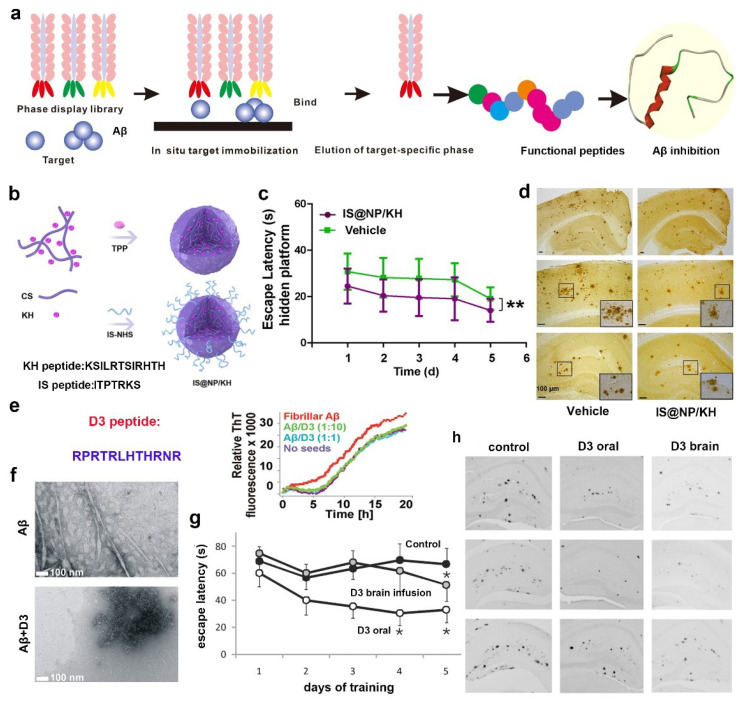
Peptides obtained using phage display and their application in AD. (**a**) General scheme of phage display technology. (**b**) Schematic illustration of the preparation process of IS@NP/KH and the sequence of KH and IS peptides. (**c**) Latency for escape to the platform through MWM experiment before and after treated with IS@NP/KH. (**d**) Immunohistochemistry staining of Aβ plaques in the brain of AD model mice, mice treated with IS@NP/KH. (**e**,**f**) D3 sequence and its effect on Aβ aggregation through TEM and ThT experiments. (**g**) Latency for escape to the platform through MWM experiment before and after treated with D3. (**h**) Immunohistochemistry staining of Aβ plaques in the brain of AD model mice, mice treated with D3 peptide. * *p* < 0.05, ** *p* < 0.01, indicating a significant difference. (**b**–**d**) Reproduced with permission from [51], Copyright 2021, American Chemical Society. (**e**–**h**) Reproduced with permission from [58], Copyright 2010, American Chemical Society.

Compelling evidence indicates that molecules, peptides, or antibodies that have a high affinity to Aβ can restrict the conformation change of Aβ and further inhibit its aggregation [41,42]. Recently, shown in Figure 2a, phage display offers an unprecedented opportunity to search the potential therapeutic peptides for various diseases based on the affinity between the resulting peptide and target molecules [43,44]. Therefore, phase display is suitable for researchers to search for peptides that have the potential to inhibit Aβ aggregation. Once obtaining the resulting peptides, affinity tests, such as isothermal titration calorimetry (ITC) [45,46], surface plasmon resonance (SPR) [47], microscale thermophoresis (MST) [48], and quartz crystal microbalance (QCM) [49], can screen the peptides and choose the most promising one for further investigation of its inhibitory effect on Aβ aggregation and the therapeutic effect on AD. In 2006, Kiessling et al. identified several Aβ-affinity peptide ligands with phage display [50]. In this experiment design, monomeric and highly aggregated Aβ were used as the target molecules, respectively. Intriguingly, it was found that the peptides screened based on the Aβ monomeric state had little effect on Aβ aggregation. In contrast, those selected based on the Aβ aggregated state increased the rate of Aβ aggregation. This study reminds us that different preparations of the targets can yield different peptides with markedly diverse effects on Aβ aggregation. Based on this situation, Gao et al. screened some Aβ42 oligomer binding peptides through phase display, and the KH (KSILRTSIRHTH) peptide was proved to have the best affinity to Aβ42 oligomer [51]. Then they combined the KH peptide and the brain-targeting peptide (IS: ITPTRKS) to develop a bifunctional nanoparticle (abbreviated as IS@NP/KH) (Figure 2b) to cross the blood-brain barrier and treat AD. The Morris water maze (MWM) test was conducted to evaluate the therapeutic effect on learning and spatial memory in mice [52], and the results indicated that IS@NP/KH can improve the cognitive performance of AD mice (Figure 2c). Additionally, the immunohistochemistry staining assay also demonstrated that IS@NP/KH can reduce Aβ plaques in the mice brain (Figure 2d). The above study showed that KH peptide screened using phase display towards Aβ42 oligomer possess therapeutic potential for AD. Moreover, the SPR-based affinity assay revealed that the peptide, which enables modulated Aβ aggregation, was correlated with its affinity to N-terminal 10 residues of Aβ (Aβ1–10). With this knowledge, phage display identified a highly specific peptide (PYRWQLWWHNWS) with a strong affinity to Aβ_1–10_ [53]. This peptide alleviated Aβ-induced PC12 cell viability and apoptosis. Then, applying the peptide to AD model mice revealed that the peptide exhibited a protective effect against Aβ-induced learning and memory deficits in mice.

However, it is worth noting that the functional peptides identified using traditional phage display are composed of natural L-amino acids. These L-peptides are prone to proteases degradation and therefore have a short half-life compared with D-peptides [54]. Additionally, D-peptides could be absorbed systemically after oral administration and are reported to be reduced in comparison to L-peptides [55,56]. Based on this situation, Kim’s groups proposed a mirror image phage display, which greatly applied phage-derived peptides in treating AD in vivo [55]. Mirror phage display allows phage display to identify peptides that consist of D-amino acids only. Based on this, Willbold’s group applied mirror phage display in diagnosing and treating AD. As shown in Figure 2e, they obtained D-enantiomeric amino acid peptide D3 through a randomized 12-mer peptide phage library and applied it into AD treatment [57,58,59,60]. ThT and TEM experiments showed that D3 could inhibit Aβ aggregation and fibril formation (Figure 2e,f). Interestingly, D3 can precipitate toxic species and converts them into nonamyloidogenic, nonfibrillar, and nontoxic aggregates without increasing the concentration of monomeric Aβ. Furthermore, the application of D3 to AD model mice followed by an MWM experiment indicated that D3 can improve the cognitive performance of AD mice (Figure 2g). Additionally, the immunohistochemistry staining assay indicated that D3 could reduce Aβ plaques in the brain (Figure 2h). In another study, they further explained the reason for the therapeutic effect of D3 on AD because it can penetrate the blood–brain barrier and thus target the Aβ through an arginine-rich motif [61]. These highlight the great potential of mirror image phages in creating various D-peptides in the treatment of AD. In future, peptides related to Aβ or Aβ oligomers could also be selected based on different phage-displayed peptide libraries, such as cyclic peptide libraries. Additionally, these peptides could be modified and then explore their inhibitory effect on Aβ aggregation and AD.

## 4. Design of Peptides Based on the Core Sequence of Aβ Fibrosis

**Figure 3:**
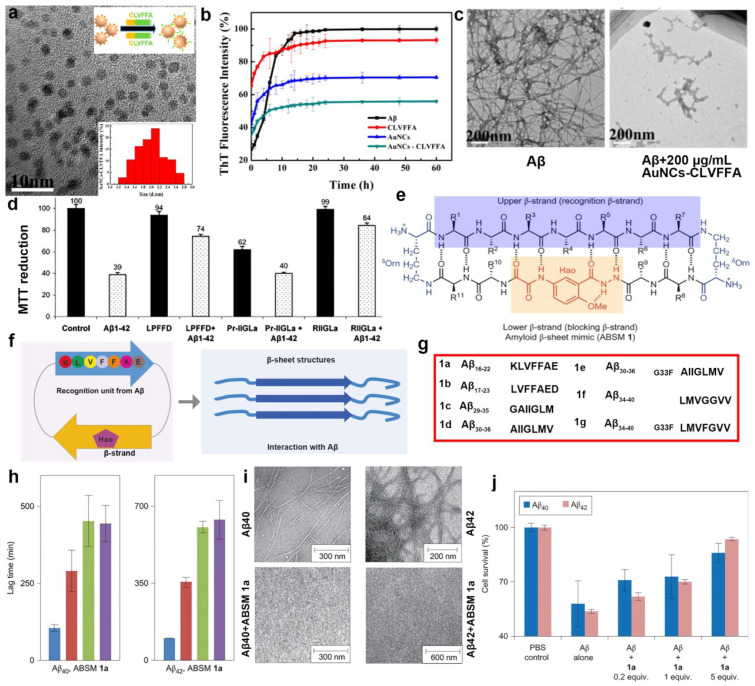
Design of peptide inhibitors based on the core sequence of Aβ fibrosis and their effect on Aβ aggregation. (**a**) The morphology of AuNCs-CLVFFA through TEM. (**b**,**c**) The effect of AuNCs-CLVFFA on Aβ aggregation through ThT and TEM experiments. (**d**) The effect of IIGL based-peptide on Aβ aggregation induced cytotoxicity through MTT assay. (**e**) Representation of ABSM 1 illustrating the upper β-strand (recognition β-strand), the δ-linked ornithine turn unit, and the Hao amino acid blocker unit. (**f**) Schematic diagram of ABSM 1 recognizing and blocking Aβ aggregation through β-sheet interactions. (**g**) Amino acid sequences from Aβ for the design of ABSMs 1a–g. (**h**,**i**) The effect of ABSM 1a on Aβ40 and Aβ42 aggregation monitored using ThT fluorescence and TEM assays. (**j**) The effect of ABSM 1a on Aβ40 and Aβ42 induced toxicity towards PC-12 cells. (**a**–**c**) Reprinted with permission from [63], Copyright 2019, American Chemical Society. (**d**) Reprinted with permission from [77], Copyright 2004, Elsevier Inc. (Amsterdam, The Netherlands). All rights reserved. (**e**,**h**–**j**) Reprinted with permission from [79], Copyright 2012, Springer Nature Limited (Berlin/Heidelberg, Germany).

The most effective treatments may be those designed to inhibit core sequences that precede protein or peptide aggregation by blocking the production of the amyloidogenic protein or peptide in the first place. Therefore, some focus on the design of peptides based on the core sequence of Aβ fibrosis. Nordstedt and coworkers showed that Aβ(16–20) (LVFFA) was able to bind to the full-length of Aβ and prevent its assembly into fibrils [62]. Based on LVFFA and gold nanoparticles, Wang et al. fabricated a hexapeptide CLVFFA modified Au clusters shown in Figure 3a. ThT and TEM results showed that AuNCs-CLVFFA inhibited Aβ40 fibrillogenesis, fibril prolongation, and mature fibril disaggregation (Figure 3b,c) [63]. Many other studies further focus on modifying KLVFF to investigate their effect on Aβ aggregation. For instance, El-Agnaf et al. designed KLVFF-derived compounds to regulate the very early aggregation of intermediates of Aβ monomers and Aβ oligomers based on the idea of adding water-soluble amino acids residues to KLVFF to aid solubility [64]. In this design, Arg functioned as a solubilizing residue at both the N- and the C-terminus of the hydrophobic peptides [65]. In addition, Gly residues, a conformationally unrestrained amino acid, were designed as spacers between Arg and the binding residues to prevent Arg from interacting with the peptide and Aβ. Finally, two peptides, OR1 (RGKLVFFGR) and OR2 (RGKLVFFGR-NH_2_), were obtained, and an NH_2_ at the C-terminus of OR2 could render the peptide less charged. Both OR1 and OR2 could inhibit Aβ fibrillation. Giralt et al. developed a peptide inhibiting Aβ toxicity in cell culture based on the KLVFF motif by adding an additional Lys to the N-terminus to increase solubility and an N-methyl-20F to block Aβ aggregation [66]. It was found that the inhibitory effect of D-KLVFFA on Aβ aggregation was more significant than L-KLVFFA [67]. D-KLVFFA modification with acetylation, amidation, and methylation also performed better in inhibiting Aβ in AD treatment [68,69,70]. These further proved that chiral peptides, particularly D-based peptides, were powerful tools for the therapeutic development of AD. Most natural peptides are cyclized. The peptides in cyclic form occupied two-thirds of the peptides approved by the FDA and EMA and have essential roles in the modern pharmaceutical industry [71,72]. For example, Kanai et al. focus on investigating the inhibitory effect of cyclic KLVFF on Aβ. The unique pharmacophore motifs comprised side-chains of Leu, Val, Phe, and Phe residues of KLVFF but not those involved with backbone amide bonds on Aβ aggregation, possessing potent activity and inhibiting Aβ aggregation [73].

Other sequences are also crucial in Aβ aggregation. Penke et al. developed an Aβ aggregation inhibitor based on the Aβ31–34 sequence IIGL, which also plays a fundamental role in Aβ aggregation and cytotoxicity [74,75,76]. The idea is the same as the abovementioned, the author linked a solubilizing amino acid residue to the original sequence to obtain RIIGL, and it can inhibit the formation of Aβ fibrils and reduce Aβ-induced cytotoxicity (Figure 3d) [77]. Additionally, Fradinger et al. prepared a series of Aβ C-terminal fragments (Aβx–42; x = 28–39). They confirmed the hypothesis that C-terminal peptides of Aβ should possess a high affinity to full-length Aβ and might disrupt Aβ oligomer formation. Cell viability assays showed that Aβ31–42 and 39–42 are the most effective inhibitors on Aβ-induced cell toxicity [78].

Based on these amyloidogenic peptides from Aβ, Nowick et al. [79] developed a new class of β-sheet macrocycles that contained a wide range of amino acid sequences from Aβ, called ABSMs, and still fold into β-sheet structures to inhibit Aβ aggregation. Among this design, for instance, shown in Figure 3e,f, ABSM1 is a 54-membered ring comprising a heptapeptide β-strand (the upper strand), one Hao unit flanked by two dipeptides (the lower strand) and two δ-linked ornithine turns. The ‘upper’ β-strand of ABSM1 incorporates different heptapeptide fragments from Aβ shown in Figure 3g. Taking ABSMs 1a as an example, ThT and TEM assays evaluating its inhibition on Aβ40 and Aβ42 aggregation found that ABSMs 1a effectively delayed the aggregation of Aβ40 and Aβ42 at substoichiometric concentrations (Figure 3h,i). Furthermore, MTT assays established that ABSM 1a reduced the toxicity induced by Aβ40 and Aβ42 in PC-12 cells (Figure 3j). These studies concluded that β-sheet macrocycles based on amyloidogenic peptides from Aβ had different inhibitory effects. Generally, designing different amyloidogenic peptides and comparing the functions of these different compositions are conductive to the treatment of AD.

## 5. Computer-Aided Peptide Inhibitors Based on the Segments and Structure of Aβ

**Figure 4:**
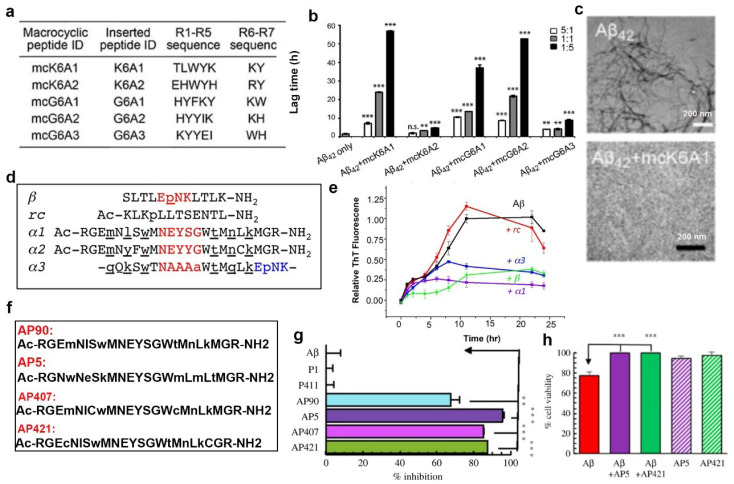
Rational design of peptides based on the structure of Aβ through computer-aided drug design. (**a**) Peptide sequences obtained RosettaDesign based on KLVFFA and GGVVIA in Aβ. (**b**) The effect of different peptides on Aβ aggregation through ThT assay. (**c**) TEM morphology before and after being treated with mck6A1. (**d**) Peptide sequences based on the Aβ oligomer structure through computer design. (**e**) The effect of peptides on Aβ aggregation through ThT assay. (**f**) The sequences of α-sheet peptides. (**g**) The effect of α-sheet peptides on Aβ aggregation through ThT assay. (**h**) The effect of α-sheet peptides on Aβ aggregation induced cytotoxicity through MTT assay. ** *p* < 0.01, *** *p* < 0.001, indicating a significant difference, n.s.: not significant. (**a**–**c**) Reproduced with permission [81], Copyright 2019, CC BY (http://creativecommons.org/licenses/by/4.0/), accessed on 4 March 2019. (**d**,**e**) Reprinted with permission from [83] in accordance with the CC-BY license. (**g**,**h**) Reprinted with permission from [84], Copyright 2022, CC BY (http://creativecommons.org/licenses/by/4.0/), accessed on 23 November 2022.

The recent development of computer-aided drug design (CADD) represents a shift from conventional design to computational pharmacological research [80]. Given the high costs and time consumption associated with the candidate drug failure in conventional pharmacology, CADD will be necessary in future. Li et al. designed a series of peptide inhibitors on Aβ42 using the RosettaDesign software package [81] based on the Aβ two segments KLVFFA and GGVVIA. GGVVIA also plays an essential role in fibril formation [82]. Therefore, preventing the self-assembly of either KLVFFA or GGVVIA may inhibit the assembly of Aβ42 fibrils. Finally, they obtained five peptide sequences, including R1–R5 (Figure 4a), and further utilized the strategy of β-sheet macrocycles [79] mentioned above to inhibit Aβ aggregation. The result indicated that the designed peptide inhibitors selectively recognize different species of Aβ, mcK6A1, mcG6A1, and mcG6A2, particularly mcK6A1, significantly inhibit the amyloid fibril formation of Aβ42 in a dose-dependent manner (Figure 4b,c). Previous studies suggested that the toxic soluble oligomeric form of different amyloid proteins shared a typical backbone conformation. Daggett et al. proposed that toxic intermediates of different amyloid proteins adopt a common, nonstandard secondary structure called an α-sheet. Accordingly, they designed some α-sheet compounds based on the conformation of Aβ oligomers via molecular dynamics simulations and confirmed the α-sheet structure of the peptides using FTIR, CD, and NMR experiments, respectively. The sequences of these peptides are shown in Figure 4d, and they have different inhibitory effects on Aβ aggregation; in particular, peptide α1 strongly inhibited Aβ aggregation than others (Figure 4e) [83]. After that, they further designed some α-sheet peptides (Figure 4f) based on the structure of the toxic oligomers in Aβ. Regarding the effect of α-sheet peptides on Aβ aggregation, ThT signal reduction showed that all the peptides, including AP90, AP5, AP407, and AP421, inhibited Aβ aggregation (Figure 4g). As shown in Figure 4h, the cell viability result proved that AP5 and AP421 were enabled to rescue the Aβ-mediated cytotoxicity [84]. These studies indicated that these α-sheet peptides produce a novel class of amyloid inhibitors that target the toxic soluble oligomeric state Aβ.

Some classification and machine learning (ML) and AI techniques (such as the Bayes model, decision tree, support vector machine, and artificial neural network) have been widely used to find anti-AD drugs. For instance, Fang et al. [85] reported a learning framework combining ML (involving vector representation of the molecular structure, pharmacophore fingerprint, and conformational isomer fingerprint) and a cross-species method for screening and verifying new effective compounds on AD. They used this learning framework to identify 18 kinds of small-molecule compounds from the natural compound library and obtained two effective AD inhibitors including kaempferol and rhapontigenin. The result found that the compounds can increase the survival and function of glutamatergic and cholinergic neurons, eliminate Aβ and tau-related pathology, and improve animal memory in the models of AD. The above study demonstrated that computational-experimental screening could help to find effective drugs. Thus, it is a potential manner of introducing these technologies to develop peptide inhibitors on Aβ. Considering the importance of Aβ in the progression of AD and the necessity of developing multitarget drugs against Aβ aggregation, CADD should draw more attention as a very powerful and effective tool in drug design of which the aim is to provide a better therapeutic effect on the symptoms and disease improvement of AD.

## 6. Conclusions and Future Perspectives

Despite significant progresses in understanding the pathobiology of AD, it still lacks effective disease-modifying therapy. Recently, peptides have garnered much attention in the drug development of neurodegenerative diseases owing to their high specificity, low toxicity, and immunogenicity, becoming potential candidate drugs for treating neurodegenerative diseases. This review summarizes various peptide-based therapies for inhibiting Aβ aggregation and treating AD. It begins with chiral amino acid- or peptide-based materials with their effect on Aβ aggregation and their great future potential as AD drugs. Then, it introduces two manners to design peptides to treat AD, including the affinity screening technique such as phase display to look for promising peptides and rational design based on the core sequence of Aβ fibrosis to obtain peptide inhibitors. After that, this review proposes that CADD is a potential technique for designing peptide inhibitors based on the segments and structure of Aβ.

However, taking advantage of these peptides or peptide-based materials to better treat AD is still a critical issue needing further consideration. AD is a complex neurodegenerative disease that not only features the deposition of Aβ plaques in the brain but also that the Aβ aggregation process could induce a series of cascade events such as mental homeostasis and immune responses. Recently, many studies have focused on combinational therapy for AD. For instance, metal ion-chelating agents, acetylcholinesterase (AChE) inhibitors, and Aβ inhibitors are integrated into one nanosystem for the treatment of AD [86]. Inspired by this insight, we could combine peptide inhibitors with anti-inflammatory factors and metal ion-chelating agents, etc., together to obtain a better therapeutic effect on AD. Additionally, the ability of inhibitors to cross the blood–brain barrier is an important factor in the treatment of AD. We believe that the extracellular vesicle (EV)-based therapeutic method has low toxicity, excellent biodegradability, low immunogenicity, and the ability to easily penetrate the blood–brain barrier [87,88,89,90]. Therefore, it is a natural, excellent example of the drug combination. Generally, peptide inhibitors and other agents could be integrated into EVs to treat AD. In addition, peptides have some limitations such as low oral bioavailability, shorter half-life, poor solubility and bioavailability, and low stability to proteolytic digestion in physiological fluids. Nonetheless, in recent years, general strategies to tackle those shortcomings have been established. In summary, peptides could be modified using both chemical and biological methods together with novel design and delivery strategies such as EVs to affect the chemical structure, or drug formulation and delivery approaches, which tackle the shortcomings without changing the structure of the peptide [91,92,93,94]. Briefly, with the development of design method of peptides, peptides-based therapies will be taken into practical clinical use quickly.

## Data Availability

Data sharing not applicable. No new data were created or analyzed in this study. Data sharing is not applicable to this article.
